# Immune Modulation with Sulfasalazine Attenuates Immunopathogenesis but Enhances Macrophage-Mediated Fungal Clearance during *Pneumocystis* Pneumonia

**DOI:** 10.1371/journal.ppat.1001058

**Published:** 2010-08-19

**Authors:** Jing Wang, Francis Gigliotti, Samir P. Bhagwat, Thaddeus C. George, Terry W. Wright

**Affiliations:** 1 Department of Pediatrics, University of Rochester School of Medicine and Dentistry, Rochester, New York, United States of America; 2 Department of Microbiology and Immunology, University of Rochester School of Medicine and Dentistry, Rochester, New York, United States of America; 3 Amnis Corporation, Seattle, Washington, United States of America; UMass Medical Center, United States of America

## Abstract

Although T cells are critical for host defense against respiratory fungal infections, they also contribute to the immunopathogenesis of *Pneumocystis* pneumonia (PcP). However, the precise downstream effector mechanisms by which T cells mediate these diverse processes are undefined. In the current study the effects of immune modulation with sulfasalazine were evaluated in a mouse model of PcP-related Immune Reconstitution Inflammatory Syndrome (PcP-IRIS). Recovery of T cell-mediated immunity in *Pneumocystis*-infected immunodeficient mice restored host defense, but also initiated the marked pulmonary inflammation and severe pulmonary function deficits characteristic of IRIS. Sulfasalazine produced a profound attenuation of IRIS, with the unexpected consequence of accelerated fungal clearance. To determine whether macrophage phagocytosis is an effector mechanism of T cell-mediated *Pneumocystis* clearance and whether sulfasalazine enhances clearance by altering alveolar macrophage phagocytic activity, a novel multispectral imaging flow cytometer-based method was developed to quantify the phagocytosis of *Pneumocystis in vivo*. Following immune reconstitution, alveolar macrophages from PcP-IRIS mice exhibited a dramatic increase in their ability to actively phagocytose *Pneumocystis*. Increased phagocytosis correlated temporally with fungal clearance, and required the presence of CD4^+^ T cells. Sulfasalazine accelerated the onset of the CD4^+^ T cell-dependent alveolar macrophage phagocytic response in PcP-IRIS mice, resulting in enhanced fungal clearance. Furthermore, sulfasalazine promoted a TH2-polarized cytokine environment in the lung, and sulfasalazine-enhanced phagocytosis of *Pneumocystis* was associated with an alternatively activated alveolar macrophage phenotype. These results provide evidence that macrophage phagocytosis is an important *in vivo* effector mechanism for T cell-mediated *Pneumocystis* clearance, and that macrophage phenotype can be altered to enhance phagocytosis without exacerbating inflammation. Immune modulation can diminish pulmonary inflammation while preserving host defense, and has therapeutic potential for the treatment of PcP-related immunopathogenesis.

## Author Summary

*Pneumocystis* is a fungal respiratory pathogen that causes life-threatening pneumonia (PcP) in immunosuppressed patients. PcP remains an infectious complication of AIDS and cancer, and is emerging in previously unrecognized clinical settings. Despite dramatic advances in health care and the availability of antibiotics to treat this infection, mortality rates have improved little over the past 25 years. T cell-mediated immunity is critical for host defense against respiratory fungal infections. However, T cells also cause PcP-related inflammation and lung injury. The results of the current study indicate that the immune response to *Pneumocystis* can be modulated to reduce tissue damaging inflammation while enhancing anti-fungal host defense. Alveolar macrophages recognize and eliminate pathogens from the lung and also regulate inflammation. We have identified alveolar macrophages as the effector cells for T cell-dependent clearance of *Pneumocystis* from the lung, and demonstrated that macrophage phenotype can be altered to enhance microbe elimination without promoting inflammatory injury. These results suggest that the effector mechanism of T cell-mediated fungal clearance is distinct from the effector mechanism of T cell-mediated lung inflammation and injury. This conceptual advance can be exploited to develop more effective therapeutic strategies to block inflammation while preserving host defense.

## Introduction

*Pneumocystis* (Pc) is an opportunistic fungal respiratory pathogen that causes life-threatening pneumonia in patients suffering from defects in cell-mediated immunity, including those with acquired immunodeficiency syndrome (AIDS) and immunosuppression secondary to chemotherapy or organ transplantation. *Pneumocystis* pneumonia (PcP) remains a leading cause of death among HIV-infected patients and a significant cause of AIDS-related mortality and morbidity [1]. For example, mortality rates of 50% or higher have been reported for AIDS patients with severe PcP [2], [3], and despite major advances in health care, the mortality associated with PcP has changed little over the past 25 years. In addition, as more powerful anti-inflammatory treatments are developed for various underlying diseases, more cases of PcP are occurring in non-HIV patients and in previously unreported clinical settings [4]–[6]. Recent studies also indicate that Pc colonization can exacerbate chronic obstructive pulmonary disease [7]. Therefore, improving the treatment of patients suffering from both HIV- and non HIV-related PcP remains a central concern of the health care community.

Although the direct pathogenic capabilities of the *Pneumocystis* organism itself are poorly understood, the role of the host's immune response as a major contributor to PcP-related lung injury has come to the forefront. In patients, the clinical severity of PcP is dictated by the degree of pulmonary inflammation, rather than by the organism lung burden [8]–[14]. Specifically, T cell and neutrophilic responses have been linked to PcP-related lung injury in patients [10], [15]. A clinical example of the immunopathogenic nature of PcP is the severe disease that has been reported in AIDS patients following successful anti-retroviral treatment [16]–[18]. This distinct clinical syndrome, termed Immune Reconstitution Inflammatory Syndrome (IRIS) or Immunorestitution Disease (IRD), occurs when CD4^+^ T cell-mediated immunity is restored following a period of immunosuppression. The recovery of immune function restores protective adaptive immunity, but does so at the cost of initiating a severe immunopathological response to a pre-existing Pc infection. An IRIS-like presentation of PcP has also been described in non-HIV infected patients following the successful tapering of steroid therapy or bone marrow engraftment [19], [20]. Importantly, patients with non-HIV presentations of PcP and IRIS seem to develop a more fulminant and acutely immunopathogenic disease than patients with a classical AIDS-related presentation in which CD4^+^ T cell function is chronically and profoundly depressed [10], [21]–[24].

The immunopathogenesis of PcP has been confirmed by controlled studies in Pc-infected severe combined immunodeficient (SCID) mice. Following adoptive transfer of normal splenocytes these mice develop disease that is pathologically similar to clinical reports of IRIS. When the host's immune system is restored, an intense T cell-mediated immune response brings about organism clearance with the undesired consequence of severe lung damage and respiratory deterioration [25]–[30]. Our laboratory has demonstrated that CD4^+^ T cells predominate in the lungs at the time of maximal injury and that depleting this population prevents the onset of acute disease [28], [31]. Other studies have demonstrated that CD4^+^ T cells are robustly pathogenic in the setting of immune recovery and PcP [26], [27], [32], [33]. While existing evidence unquestionably demonstrates that T cell responses are directly involved in both the clearance of Pc and the generation of immune-mediated lung injury, the specific downstream effector mechanisms have not been elucidated. Alveolar macrophages (AMs) are likely involved in both of these processes, but their *in vivo* role remains incompletely defined.

Sulfasalazine (SSZ) is a potent anti-inflammatory drug commonly used to treat the inflammatory consequences of Crohn's disease and Rheumatoid Arthritis [34]–[36]. SSZ modulates immune responses by altering macrophage and T cell responses [37]–[39]. Many effects of SSZ are related to its function as a potent inhibitor of NF-κB [39], [40], a signaling pathway that is important for inflammatory responses to Pc [38], [41], [42], [43]. Therefore, we hypothesized that the potent immunomodulatory properties of SSZ could alleviate lung injury and improve outcome in a mouse model of PcP-related IRIS. SSZ was highly effective for attenuating the immune-mediated lung injury associated with PcP, with the unexpected finding that SSZ also accelerated fungal clearance. Moreover, we developed a multispectral imaging flow cytometer-based method to assess Pc phagocytosis *in vivo*. Using this technology we established that the macrophage is the downstream effector for CD4^+^ T cell-dependent clearance of Pc from the lung, and that SSZ enhances clearance by promoting AM phagocytosis.

## Results

### Sulfasalazine markedly reduces the severity of PcP

**Figure 1:**
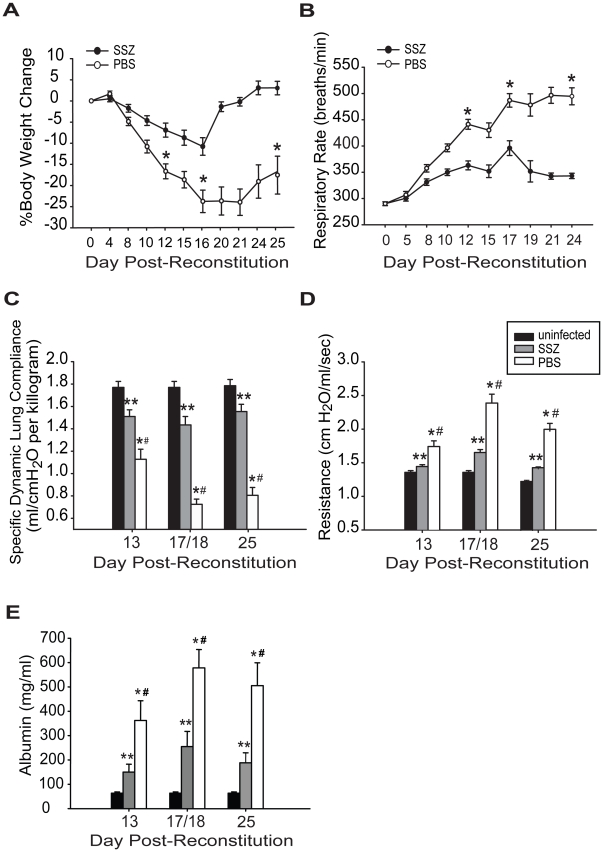
SSZ treatment reduces the severity of PcP-related IRIS. Pc-infected SCID mice were immunologically reconstituted and then treated with PBS or SSZ beginning at day 1 post-reconstitution. (A and B) Body weight and respiratory rates were monitored non-invasively. (C, D, E) Dynamic lung compliance, lung resistance and BAL albumin content were measured at 13, 18, and 25 days post-reconstitution. Values are mean ±1 standard error measurement (SEM) (n≥15 for day 13, n≥37 for day 18, and n≥15 for day 25). ** and #, P<0.05 as compared to uninfected mice at the same time point. *, P<0.05 as compared to SSZ treated mice at the same time point. Mean represents combined data from eight independent experiments.

SSZ is used clinically to treat conditions in which inflammation is integral to pathogenesis. To test the efficacy of SSZ for reducing the severity of PcP-related IRIS, infected SCID mice were immunologically reconstituted with wild type splenocytes, and then treated with either SSZ or PBS vehicle beginning at day 1 post-reconstitution (PR). Respiratory rates and body weights were monitored non-invasively, and dynamic lung compliance and resistance were measured at 13, 18 and 25 days PR. These times correspond to the early, peak, and resolution phases of PcP in this model. As expected, the PBS-treated mice with IRIS exhibited progressive disease that was characterized by dramatic weight loss and elevated respiratory rates. These mice lost an average of 17±2% body weight by day 12, and 24±3% by day 16 PR. Thereafter, the mice began to gain weight coincident with the resolution of disease (Figure 1A). These mice also exhibited elevated respiratory rates, which increased dramatically to an average of 441±9 respirations per minute at day 12 and 487±13 respirations per minute by day 17 PR (Figure 1B). In contrast, the SSZ-treated mice exhibited only slight variations in body weight and respiratory rate over this same period, and had a generally healthy appearance.

Direct pulmonary function measurements were taken at days 13, 18, and 25 PR. Dynamic lung compliance and lung resistance are derived from pressure and volume measurements recorded on live ventilated mice. Lung compliance is a measure of the lungs ability to stretch during the respiratory cycle, and is considered a measure of alveolar health. Mice with PcP have reduced compliance compared to healthy mice, indicating that the lungs are less elastic and generate greater pressure during respiration. Lung resistance is a measure of air flow limitation to and from the gas exchange surface, and can be negatively affected by airway and alveolar inflammation. Mice with PcP have increased resistance compared to healthy mice. Both of these measures are good indicators of the severity of PcP. PBS-treated mice with IRIS developed a drastic deterioration of pulmonary function over the course of the study. A severe reduction in dynamic lung compliance was observed by day 13 PR, and by day 18 these mice demonstrated a 59% deficit in lung compliance (Figure 1C). In contrast, the SSZ-treated mice suffered only a 19% reduction in lung compliance over this same period, and recovered to nearly normal pulmonary function by day 25 PR. Similarly, a dramatic difference in resistance values was observed between the SSZ- and PBS-treated IRIS mice (Figure 1D). The SSZ-treated mice exhibited significantly lower lung resistance than the vehicle group at all time points, supporting the conclusion that SSZ decreases the magnitude of the pulmonary function deficits associated with PcP, and attenuates overall disease severity.

To determine the effect of SSZ treatment on epithelial damage and alveolar permeability during PcP, albumin content was measured in the bronchoalveolar lavage (BAL) fluid. Elevated levels of albumin in the BAL fluid indicates damage to the tight junctions between alveolar epithelial cells and serves as a marker for the severity of PcP. PBS-treated mice with PcP had significantly elevated albumin levels on days 13 and 18 PR as compared with normal uninfected mice (Figure 1E). In contrast, the SSZ-treated mice had lower albumin levels than PBS-treated mice at both time points (Figure 1E). While albumin levels returned toward baseline in both groups by day 25, they remained significantly lower in the SSZ-treated mice. These data demonstrate that SSZ attenuates damage to the alveolar-capillary barrier, which contributes to the preservation of pulmonary function during PcP.

### Sulfasalazine reduces pulmonary inflammation during PcP-related IRIS

Total cell counts, differentials, and flow cytometry were also performed on BAL cells from experimental mice. PBS-treated IRIS mice had significantly elevated numbers of total BAL cells compared to SSZ-treated mice at all time points (Table 1). Differential staining revealed that the reduced number of cells in SSZ-treated mice relative to PBS-treated mice was mainly a reflection of fewer lymphocytes and neutrophils at all time points (Table 1). As neutrophil numbers are predictive of disease severity, fewer neutrophils in SSZ-treated mice was also an indicator of less severe disease. Despite a reduction in total cells, it was notable that SSZ-treated mice had more AMs than PBS-treated mice at day 18. Flow cytometry analyses revealed that SSZ-treated mice had fewer CD4^+^ and CD8^+^ T cells than PBS-treated mice at 13 and 18 days PR. By day 25 PR the reduced BAL cells in SSZ-treated mice was mainly a reflection of reduced CD4^−^/CD8^−^ lymphocytes (possibly B cells) and neutrophils.

**Table 1:** Cellular composition of BAL fluid* from SSZ- and PBS-treated IRIS mice.

Cell Type	Day13^a^		Day18		Day25	
	SSZ	PBS	SSZ	PBS	SSZ	PBS
Total cells (10^5^)	1.99±0.21	11.2±1.97^b^	7.97±1.06	17.9±3.11^c^	5.06±0.35	13.0±2.26^d^
Macrophages (10^5^)	0.93±0.21	1.98±0.31^e^	5.07±0.46	3.90±0.86^f^	3.65±0.23	4.30±0.67
Lymphocytes (10^5^)	0.27±0.08	2.81±0.63^g^	1.82±0.46	7.24±1.36^h^	1.13±0.24	7.38±1.51^i^
CD4^+^ T cells (10^5^)	0.04±0.01	0.39±0.1^j^	0.47±0.14	0.89±0.17	0.16±0.11	0.25±0.06
CD8^+^ T cells (10^5^)	0.11±0.06	0.79±0.2^k^	0.64±0.34	1.66±0.41^l^	0.61±0.37	0.49±0.26
Neutrophils (10^5^)	0.66±0.11	6.10±1.55^m^	0.81±0.27	6.36±1.05^n^	0.1±0.04	1.09±0.61^o^

*: BAL fluid was isolated from one-half of the lung.

a days post-immune reconstitution.

**b–o:** P<0.05 as compared with uninfected mice and SSZ-treated mice at the same time point, n≥18/time point/group, data is pooled from three independent experiments.

**Figure 2:**
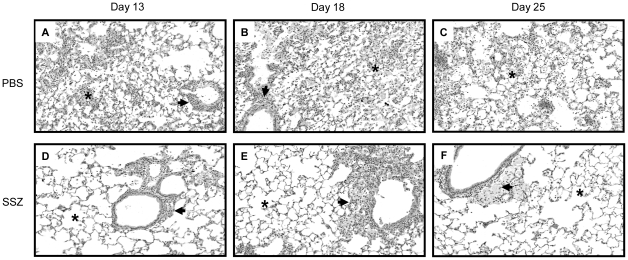
SSZ reduces pulmonary inflammation during PcP-related IRIS. Pc-infected SCID mice were immunologically reconstituted, and then treated with PBS (Panels A–C) or SSZ (Panels D–F) beginning at day 1 post-reconstitution. Lung sections from mice at days 13 (A, D), 18 (B, E) or 25 (c, F) post-reconstitution were stained with Hematoxylin and Eosin. Representative pictures were taken by microscopy under 40× magnification. Arrows denote peribronchiolar regions in each section, while asterisks denote alveolar regions.

Histological examination of hematoxylin and eosin stained lung sections showed that the PBS-treated mice exhibited a more intense, wide-spread inflammatory response than the SSZ-treated mice. Pulmonary inflammation in PBS-treated mice was characterized by the accumulation of monocytic and polymorphonuclear aggregates throughout the alveoli, although the inflammation appeared less intense by day 25 (Figure 2A–C). In contrast, evidence of inflammation in the SSZ-treated mice at days 13 and 18 PR was mostly limited to perivascular and peribronchial regions, and the alveoli were mainly free from cellular infiltrates (Figure 2D, E). Furthermore, the inflammation was almost completely resolved by day 25 in the SSZ-treated mice (Figure 2F).

### Sulfasalazine inhibits lung chemokine and cytokine production during PcP

**Figure 3:**
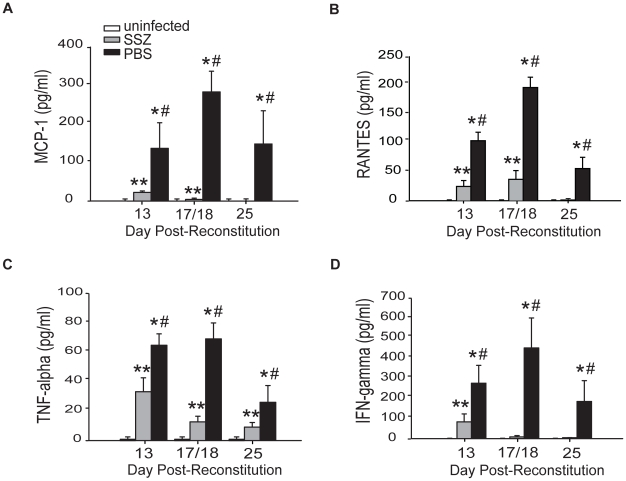
SSZ reduces inflammatory chemokine and cytokine production during PcP-related IRIS. Pc-infected SCID mice were immunologically reconstituted, and then treated with PBS or SSZ. (A, B, C, and D) MCP-1, RANTES, TNF-α, and IFN-γ levels were measured in the BAL fluid. Values are mean ±1 SEM (n≥15/time point/group). ** and #, P<0.05 as compared to uninfected mice at the same time point. *, P<0.05 as compared to SSZ treated mice at the same time point. Mean represents combined data from three independent experiments.

Elevated cytokine production is a characteristic of PcP-related inflammation. To determine whether the reduced severity of PcP observed in SSZ-treated mice was associated with blunted chemokine and cytokine production in the lung, MCP-1, RANTES, TNF-α, and IFN-γ levels were measured in the BAL fluid. Cytokine and chemokine levels were elevated above control levels in the lungs of PBS-treated IRIS mice, consistent with the physiological and histological data showing severe inflammatory disease. In contrast, lung levels of MCP-1, RANTES, TNF-α and IFN-γ in the SSZ-treated group were all significantly lower than the PBS-treated group at all time points (Figure 3A–D). Reduced cytokine levels in the SSZ-treated mice were associated with reduced inflammatory disease in these mice.

### Sulfasalazine enhances the host's ability to clear the Pc infection

**Figure 4:**
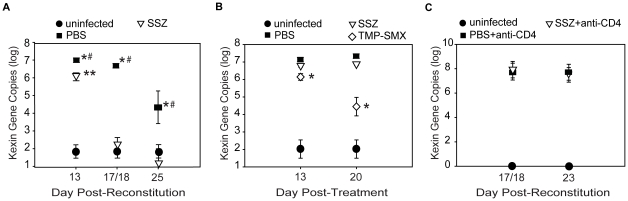
SSZ enhances Pc clearance through a CD4-dependent mechanism. (A) Pc burden in immune reconstituted SCID mice that received SSZ- (∇) or PBS (▪). Values are mean ±1 SEM (n≥15 for day 13, n≥37 for day 18, and n≥15 for day 25). ** and #, P<0.05 as compared to uninfected mice at the same time point. *, P<0.05 as compared to SSZ treated mice at the same time point. Mean represents combined data from eight independent experiments. (B) Pc burden in non-reconstituted SCID mice after 13 and 20 days of SSZ (∇), PBS (▪), or TMP-SMX (◊) treatment. Values are mean ±1 SEM (n≥11 for day 13, and n≥17 for day 20). *, P<0.05 as compared to SSZ and PBS treated mice at the same time point. Mean represents combined data from three independent experiments. (C) Pc burden in immune reconstituted, CD4-depleted SCID mice receiving SSZ (∇) or PBS (▪). Values are mean ±1 SEM (n≥8 for day 18 and n≥14 for day 23). Mean represents combined data from four independent experiments.

To study the effect of SSZ on fungal clearance, the lungs of experimental mice were examined for Pc burden using real-time PCR. Both PBS- and SSZ-treated mice had significant lung Pc burdens on day 13 PR (Figure 4A). Although the PBS-treated mice showed little reduction in Pc burden by day 18 (log 6.77±0.07), the SSZ-treated mice exhibited a 4-log reduction between days 13 and 18 PR (log 2.25±0.42) (Figure 4A). By day 25 PR, Pc burdens in the PBS-treated IRIS mice were reduced by an average of 2 logs, while Pc was undetectable in nearly all individual SSZ-treated animals (Figure 4A). The above data were combined from 8 independent experiments. The enhanced clearance of Pc in SSZ-treated mice was confirmed by enumerating Pc cysts in the lung homogenates with Gomori's methenamine silver stain (data not shown). Therefore, SSZ accelerates the fungal clearance kinetics in this model.

### Enhanced Pc clearance in SSZ-treated mice requires CD4^+^ T cell mediated immunity

Adaptive immunity is critical for the clearance of Pc from the lungs. To determine whether the mechanism by which SSZ enhances Pc clearance requires the adaptive immune system, non-reconstituted Pc-infected SCID mice were administered either PBS vehicle, SSZ (200 mg/kg/day), or trimethoprim sulfamethoxazole (TMP-SMX) (16 mg/kg/day). As expected, PBS-treated SCID mice had high Pc burdens at both 13 and 20 days post-treatment (Figure 4B). Similarly, the SSZ-treated group also had high Pc burdens at both time points and did not clear the Pc infection (Figure 4B), demonstrating that SSZ had no direct Pc killing effect. In contrast, the TMP-SMX-treated group showed reduced Pc burden at both time points (Figure 4B). The role of CD4^+^ T cells in host defense against Pc has been well-documented. To determine whether the mechanism of SSZ-enhanced clearance is CD4^+^ T cell-dependent, Pc-infected SCID mice were immune reconstituted and then CD4^+^ T cell depleted. The Pc burdens of PBS- and SSZ-treated mice were determined at days 18 and 25 PR. Neither PBS- nor SSZ-treated mice cleared the Pc infection in the absence of CD4^+^ T cells (Figure 4C). Thus, the mechanism by which SSZ enhances Pc clearance requires a CD4^+^ T cell response. These data demonstrate that SSZ does not have direct anti-Pc activity, and that SSZ-enhanced clearance arises from modulation of the CD4^+^ T cell-mediated immune response.

### Sulfasalazine enhances CD4^+^ T cell-dependent AM phagocytosis

**Figure 5:**
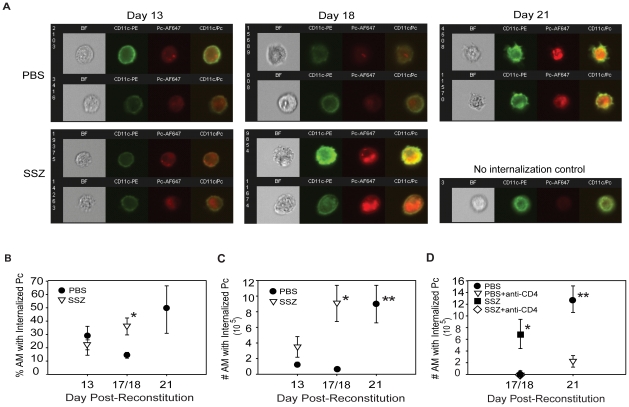
SSZ enhances CD4+ T cell-dependent AM phagocytosis of Pc. BAL cells were collected from PBS- and SSZ- treated mice at day 13, 17 or 18 (17/18) and 21 post-reconstitution. Cells were stained with antibodies specific for CD11c (green) and Pc (red). (A) Imaging flow cytometry was used to quantify Pc internalization by AMs. Representative images of brightfield (BF), CD11c, Pc, and merged CD11c/Pc are shown for SSZ or PBS-treated mice following immune reconstitution. The no internalization control is a representative CD11c^+^ cell without internalized Pc. ImageStream IDEAS software was used to quantify (B) percentile and (C) number of AMs with Internalized Pc in immune reconstituted SCID mice treated with SSZ (∇) or PBS (•). Values are mean ±1 SEM (n≥5 for day 13, n≥9 for day 17/18, and n≥9 for day 21). *, P<0.05 as compared to PBS-treated mice at the same time point. **, P<0.05 as compared to PBS-treated mice at day 17/18. Mean represents combined data from three independent experiments. (D) Quantification of *in vivo* Pc phagocytosis in the presence or absence of CD4^+^ T cells. Reconstituted SCID mice were treated with PBS- (•), PBS + anti-CD4 (∇), SSZ (▪), or SSZ + anti-CD4 (⋄). Phagocytosis was measured at 17/18 or 21 days post-reconstitution. Values are mean +1 SEM (n≥5 for day 13, n≥9 for day 17/18, and n≥8 for day 21). *, P<0.05 as compared to PBS treated, PBS + anti-CD4-treated, and SSZ + anti-CD4-treated mice at the same time point. **, P<0.05 as compared to PBS + anti-CD4-treated mice at the same time point. Mean represents combined data from three independent experiments. Note that values for PBS treated, PBS + anti-CD4-treated, and SSZ + anti-CD4-treated mice at day 17/18 are all indistinguishable, and the symbols overlap.

Although it has been inferred that AMs are responsible for CD4^+^ T cell-mediated clearance of Pc, there is little *in vivo* evidence to support this. Therefore, a multispectral imaging flow cytometry-based method was developed to quantify AM phagocytosis of Pc *in vivo*. The Amnis ImageStream, which combines digital imaging with traditional flow cytometry, allowed for dual staining of AM surface markers and internalized Pc. Because of the large number of cells that can be rapidly evaluated, a quantitative assessment of AM internalization of Pc was generated. This method was used to assess AM phagocytic activity in SSZ and PBS treated animals at various time points. CD11c was used as a marker for AMs, and a pool of five monoclonal antibodies that recognize the surface of mouse Pc were used to determine internalization of Pc relative to the AM. Figure 5 (Panel A) shows representative images of brightfield (BF), CD11c (green), Pc (red), and CD11c-Pc merged. The no internalization control is a representative cell with CD11c signal, but no Pc internalization (Figure 5A). Control staining was performed without inclusion of anti-Pc antibodies. As expected, no red signal was observed in these cells. The flow cytometer data were then quantified for AMs from individual SSZ or PBS treated mice at days 13, 17/18, and 21 PR. The percentage of AMs with internalized Pc in SSZ-treated mice was significantly higher than in PBS-treated mice at day 17/18 PR (Figure 5B). Furthermore, when the absolute number of AM with internalized Pc was determined, the difference was even more striking. SSZ-treated mice had a 9-fold greater number of AM with internalized Pc than PBS-treated mice at days 17/18 PR (Figure 5C). Importantly the number of AM with internalized Pc increased dramatically in PBS-treated mice at day 21 PR, coincident with Pc clearance and recovery from infection. These data indicate that AM-mediated clearance also occurs in PBS-treated mice, but that SSZ treatment accelerates this T cell-dependent process.

A CD4^+^ T cell response was required for Pc clearance in both PBS and SSZ-treated animals. To determine whether CD4^+^ T cells are required for AM phagocytosis of Pc, infected SCID mice were immune reconstituted and given SSZ, SSZ plus anti-CD4 antibody, PBS, or PBS plus anti-CD4 antibody. As expected, SSZ-treated mice exhibited a large increase in AM phagocytosis of Pc at day 17/18 PR (Figure 5D), and also exhibited significant fungal clearance at this time with an average Pc burden of log 2.8±1.1 (as assessed by kexin gene copies). In contrast, SSZ-treated mice that were depleted of CD4^+^ T cells displayed nearly undetectable levels of AM phagocytosis of Pc (Figure 5D), and maintained high average Pc lung burdens of log 7.5±0.1 (p<0.05 compared to SSZ-treated mice). Likewise, PBS-treated animals displayed increased AM phagocytosis at day 21 PR, but phagocytosis was nearly undetectable in the absence of CD4^+^ T cells (Figure 5D). Consistent with these results, PBS-treated mice had lower average Pc burdens than the PBS plus anti-CD4 group (log 6.7±0.5 versus log 7.6±0.01; p<0.05). Together, these data demonstrate that AM phagocytosis is the effector mechanism for CD4^+^ T cell-dependent clearance of Pc from the lung, and that SSZ alters the lung immune response in a manner that accelerates the macrophage-mediated phagocytosis of Pc.

**Figure 6:**
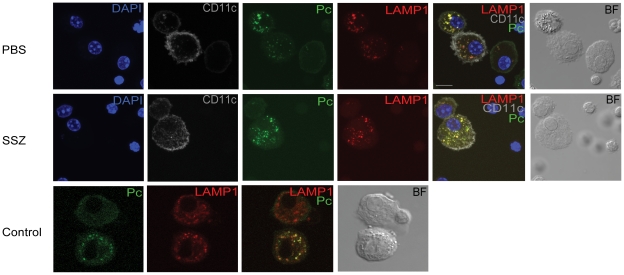
Co-localization of internalized Pc with the lysosomal protein LAMP-1. BAL cells were collected from PBS-treated mice at day 21 and SSZ-treated mice at day 17 post-reconstitution. Cells were stained with antibodies specific for CD11c (gray), Pc (green) and LAMP-1 (red). Nuclei were stained with DAPI (blue). Control image compares two AM in the same field; one with internalized Pc and one without. The scale bar represents 10 µM. Images are representative of two independent experiments.

In order to validate the ImageStream data, confocal microscopy was used to confirm the internalization and localization of Pc within AMs. BAL cells were recovered from PBS- and SSZ-treated mice at time points when AMs are actively phagocytosing Pc. Based on the data in Figure 5, the time points chosen were day 21 PR for PBS-treated mice, and day 17 PR for SSZ-treated mice. Cells were stained with DAPI (blue), anti-Pc antibody (green), anti-CD11c antibody (gray), and anti- lysosomal-associated membrane protein-1 (LAMP-1) antibody (red). LAMP-1 is a lysosome-specific protein, and was used to co-localize Pc staining to the phagolysosome inside AMs. As shown in Figure 6, we found that Pc signal co-localized with LAMP-1 signal inside CD11c^+^ AMs from both PBS-(Day 21) and SSZ-(Day 17) treated animals. The “Control” Panel shows a direct comparison between an AM with Pc and one without Pc next to each other in the same field. The cell without Pc (top) shows more diffuse LAMP-1 staining, while the bottom cell shows more focal LAMP-1 staining that co-localizes with Pc signal. This panel demonstrates differential staining and specificity of LAMP-1 and Pc antibodies. These data validate the quantitative ImageStream data as a measure of Pc internalization.

### Sulfasalazine promotes a TH2 lung cytokine environment and alternative activation of alveolar macrophages

**Figure 7:**
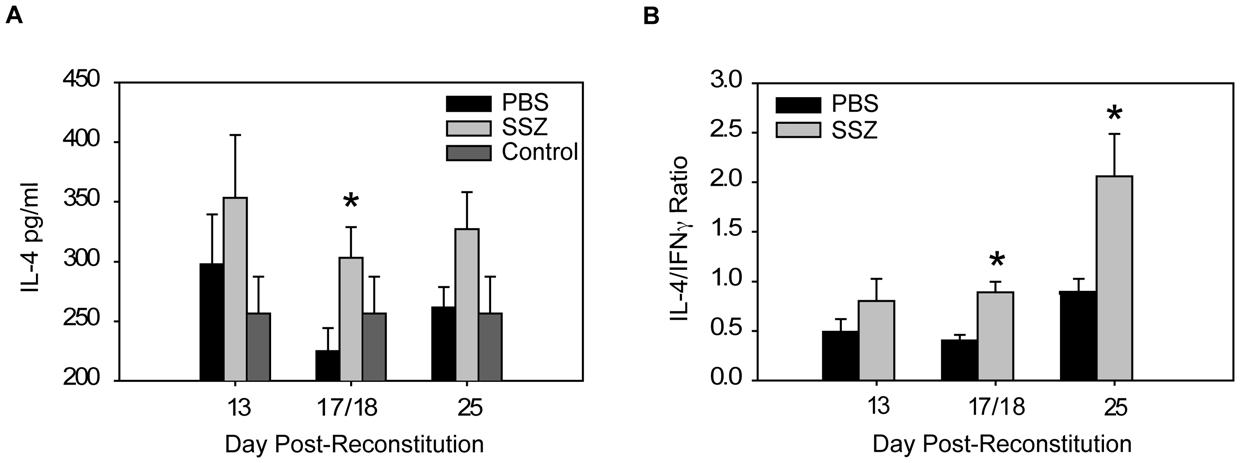
SSZ promotes a TH2 cytokine environment during PcP-related IRIS. Pc-infected SCID mice were immunologically reconstituted, and then treated with PBS or SSZ. (A) IL-4 levels and (B) IL-4/IFN-γ ratios were measured in the lung homogenates of experimental mice at indicated time points. Values are mean ±1 SEM (n≥10/time point/group). *, P<0.05 as compared to PBS treated mice at the same time point. Mean represents combined data from five independent experiments.

Macrophages are immune effector cells for T cell-dependent responses, and distinct macrophage phenotypes with differential effects on host defense and inflammation have been identified [44], [45]. Classically activated macrophages (CAM) are induced by TH1 cytokines, produce inducible nitric oxide synthase (INOS), and are pro-inflammatory. In contrast, alternatively activated macrophages (AAM) are induced by TH2 cytokines, produce arginase (ARG), are highly phagocytic, and produce anti-inflammatory mediators. Our studies have demonstrated that SSZ has profound effects on CD4^+^ T cell-dependent macrophage responses to Pc. Therefore, to determine whether SSZ alters PcP-related IRIS by modulating the polarity of the T helper response and subsequent AM effector phenotype, TH cytokine levels and macrophage activation state were assessed in experimental mice. SSZ treatment caused a dramatic decrease in lung IFN-γ production (Figure 3), with a concomitant increase in lung IL-4 production compared to PBS-treated mice (Figure 7A). Thus SSZ produced a significant shift in the IL-4 to IFN-γ ratio in the lungs (Figure 7B), effectively creating a pro-TH2 lung cytokine environment. In contrast, PBS-treated IRIS mice exhibited a pro-TH1 lung cytokine environment.

**Figure 8:**
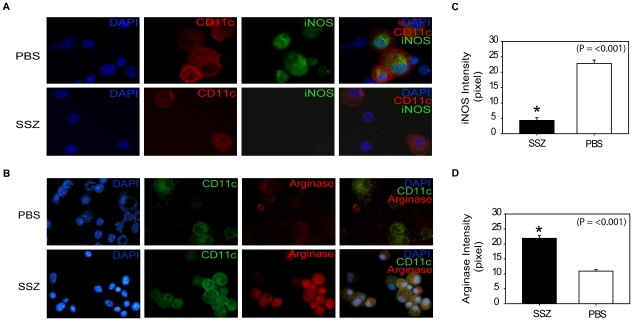
SSZ promotes an alternatively activated macrophage phenotype during PcP-related IRIS. BAL cells were collected from PBS- and SSZ- treated mice at day 13 post-reconstitution. (A) Cells were stained with antibodies specific for CD11c (red) and iNOS (green). (B) Cells were stained with antibodies specific for CD11c (green) and Arginase (red). Nuclei were stained with DAPI (blue). Images shown are 40× magnification. ImageJ software was used to quantify the mean fluorescent intensity of iNOS (C) and Arginase (D) staining of CD11c positive cells. Values are mean ±1 SEM (Panel A, n = 23 for PBS and n = 58 for SSZ; Panel B, n = 44 for PBS and n = 105 for SSZ). *, P<0.05 as compared to PBS treated mice at the same time point. Mean represents combined data from two independent experiments.

To determine whether SSZ treatment altered AM phenotype, AMs from SSZ- and PBS-treated IRIS mice at day 13 PR were assessed for INOS and ARG protein expression. Because other cell types were present in the BAL fluid from mice with PcP, CD11c was used as a surface marker for AMs. CD11c positive AMs from PBS-treated IRIS mice stained intensely for INOS (Figure 8A), but weakly for ARG (Figure 8B). In contrast, CD11c positive AMs from SSZ-treated mice stained weakly for INOS (Figure 8A), but intensely for ARG (Figure 8B). Measurement of mean fluorescent intensity of INOS and ARG staining in CD11c positive cells was used to quantify the differential expression of CAM and AAM markers in SSZ- and PBS-treated mice (Figure 8C, D). These data demonstrate that SSZ promotes alternative activation of AMs, which is associated with reduced immunopathogenesis but enhanced phagocytosis and accelerated fungal clearance.

## Discussion

IRIS is a clinical manifestation of PcP that occurs in certain patients when cell-mediated immunity is restored following a period of immune suppression and infection [16], [18], [19], [46], [47]. The resulting acute pulmonary inflammatory response restores host defense, but also produces severe pathology. The current study demonstrates that modulation of the immune response dramatically reduces the severity of PcP-related IRIS. The SSZ-mediated reduction in physiological impairment was associated with abrogated cytokine responses, reduced immune cell recruitment to the lung, and reduced histological evidence of inflammation. Unexpectedly, SSZ did not impair, but actually enhanced the host's ability to clear the Pc infection, indicating that the immune pathways leading to injury are at least partly independent of the pathways leading to clearance. Macrophages are equipped to recognize and eliminate pathogens as well as promote or resolve inflammation. To test the hypothesis that SSZ enhances Pc clearance via downstream functional alteration of AMs, a multispectral imaging flow cytometry-based method was developed to assess and quantify the *in vivo* Pc phagocytic activity of AM. This technology demonstrated that AM are effector cells for CD4^+^ T-cell mediated Pc clearance, and that SSZ enhances clearance by accelerating the AM phagocytic response. Subsequent studies found that SSZ promotes alternative activation of AMs, which is associated with reduced immunopathogenesis, but enhanced phagocytosis and fungal clearance. These data demonstrate that AM can be phenotypically modified to enhance fungal phagocytosis and clearance without ehnancing their pro-inflammatory properties, and also provide *in vivo* evidence that macrophage phagocytosis is the mechanism of CD4^+^ T cell-dependent Pc clearance from the lung.

SSZ is a clinically important immunomodulatory therapy for inflammatory diseases such as inflammatory bowel disease and rheumatoid arthritis [34]–[36]. Most of the beneficial effects of SSZ are attributed to its function as a potent inhibitor of NF-κB. SSZ directly inhibits the activity of Inhibitor of κB Kinase (IKK), effectively preventing downstream κB-dependent transcriptional events [39], [40]. Recent clinical studies have confirmed that the beneficial effects of SSZ in patients with ulcerative colitis are in fact related to inhibition of NF-κB activation in the mucosa, which results in reduced cytokine production, and less severe inflammation [48]. In addition to IKK inhibition, other mechanisms of SSZ action have been described. SSZ inhibits 5-aminoimidazole-4-carboxamidoribonucleotide transformylase causing the release of adenosine [49], [50], which controls inflammation at least partly through inhibition of NF-κB signaling [51]. Other investigators found that SSZ may alleviate inflammation in a mouse model of inflammatory bowel disease by interacting with PPAR (peroxisome proliferator-activated receptor) nuclear receptors [52]. It is noteworthy that a common mechanism of all of these interactions is related to NF-κB inhibition, and it seems likely that SSZ-mediated blockade of NF-κB is central to the beneficial effects observed in our model. In fact, a highly specific IKK inhibitor, BMS-345541, mimicked the beneficial effects of SSZ on PcP-related lung injury and pulmonary dysfunction, suggesting that NF-κB plays an important role in the immune cascade leading to the development of PcP. However, BMS-345541 did not enhance pathogen clearance. Therefore, SSZ may have other, IKK-independent, immunomodulatory properties that account for the beneficial effects on AM phagocytosis and pathogen clearance.

The beneficial action of SSZ may result from its effects on a single cell type, or more likely, from its combined effects on several cell types that contribute to injury and disease. Potential lymphocyte targets of SSZ include CD4^+^, CD8^+^, and B lymphocytes. SSZ has pro-apoptotic effects on activated T cells [37], which may contribute to the reduced T cell numbers and inflammation found in PcP-related IRIS. SSZ also influences macrophage function through the induction of apoptosis, as well as alteration of macrophage inflammatory responses [53], [54]. SSZ blocked TNF production and also abrogated IL-12 expression and NO production by stimulated macrophages [55]. Modification of macrophage IL-12 may represent a mechanism by which SSZ alters the nature of the T cell response during IRIS. NF-κB is also involved in pulmonary epithelial cell inflammatory responses to Pc [38], [41], [56], providing another potential target for the action of SSZ. While the immunopathology associated with PcP and IRIS requires T cells, other cell types likely contribute to the overall disease process, and therefore the effectiveness of SSZ reported here likely results from multiple points of action.

Our studies have found that SSZ produces a TH2 shift in the lung cytokine environment during PcP-related IRIS, and that this shift is reflected in the phenotype of alveolar macrophages. TH2 cytokines lead to alternative activation of macrophages, and consistent with a TH2 cytokine shift we found that AMs isolated from SSZ-treated mice express high levels of the AAM marker ARG, but low levels of the CAM marker INOS (Figure 8). In contrast, AMs from PBS-treated IRIS mice display a CAM phenotype with high expression of INOS. It is notable that despite a well-documented role for INOS in host defense, these data suggest that enhanced Pc phagocytosis in SSZ-treated mice is associated with an alternatively activated AM phenotype with low expression of INOS. Based on our results, we believe that increased phagocytosis by alternatively activated macrophages is the mechanism of enhanced Pc clearance. However, a role for INOS in Pc killing cannot be excluded. Although we observe less staining in AMs from SSZ-treated mice, they are not totally devoid of INOS protein. More extensive studies will be required to determine the contribution of INOS in this model.

Although we have not demonstrated that the TH2 shift is solely responsible for the beneficial effects of SSZ during PcP, it is possible that TH2 cytokines acting through AAM effectors can increase fungal clearance while reducing immunopathogenesis. For example, TH2 cytokines enhance macrophage phagocytosis of *Candida albicans* by inducing macrophage expression of mannose receptor (MR) [57], [58] and dectin-1 [59]. These pattern recognition molecules are markers of the AAM phenotype, and have known roles in anti-fungal host defense. Similarly a TH2 shift in SSZ-treated mice could enhance phagocytosis of Pc by eliciting AAM with increased expression of MR and dectin-1. In addition, a TH2 shift may also attenuate the immunopathogenesis of PcP by reducing the production of pro-inflammatory TH1 cytokines, while enhancing production of anti-inflammatory TH2 cytokines. Elevated lung levels of the TH1 cytokines TNF-α and IFN-γ are associated with PcP-related lung injury and respiratory impairment [28]. In contrast, TH2-derived AAMs produce the potent anti-inflammatory cytokines IL-10 and TGF- β [44], which can dampen inflammatory responses and may contribute to the reduced inflammation and injury in SSZ-treated mice. Importantly, the anti-inflammatory potential of AAMs has been established *in vivo* by studies showing that the adoptive transfer of *in vitro* programmed AAMs attenuates immunopathogenesis in mouse models of inflammatory disease [60]. Although these findings are consistent with a SSZ-induced shift in the polarity of the T cell response, further studies are required to establish whether TH2 cytokines and alternative activation of AMs are directly responsible for the beneficial effects of SSZ during PcP.

Clinical studies have found that the severity of PcP correlates with the degree of pulmonary inflammation, but not with organism burden [9]–[14]. Controlled animal studies support these clinical observations, and have provided direct evidence that the immune response is a major pathogenic component of PcP [26], [28]–[31], [61]. Consequently, antibiotic treatment does not always produce rapid improvement of patients with severe PcP, because organisms and antigen may continue to drive the pathological immune response. The efficacy of SSZ in dramatically attenuating the severity of PcP supports the contention that adjunctive immunomodulatory therapy that target the T cell response is critical to optimal treatment of patients. Currently, adjunctive corticosteroids are commonly used for the clinical treatment of PcP. The broad anti-inflammatory and immunosuppressive properties of steroids are presumed to provide benefit, but concrete evidence that steroids improve survival is lacking. Our group has recently published a study demonstrating that specific disruption of the T cell response to Pc with anti-CD3 antibody has beneficial effects in a mouse model of PcP-related IRIS [62]. While both SSZ and anti-CD3 altered the T cell response to Pc and reduced immunopathogenesis, they produced differential outcomes with respect to fungal clearance. Anti-CD3 produced a profound inhibition of T cell responses which reduced disease, but also prevented the clearance of Pc from the lung. In contrast, SSZ dampened PcP-related immunopathogenesis without suppressing TH responses to a degree that prevented eradication of the organism. SSZ not only reduced T cell-mediated inflammation, but altered the nature of the T cell response by promoting TH2 lung cytokine environment and alternative activation of macrophages. It is likely that the preservation of TH2 responses combined with a shift in the polarization of AMs in SSZ-treated mice is responsible for the differential effects of SSZ and anti-CD3.

Another important aspect of our work is the development of a multispectral imaging flow cytometer-based method to assess the *in vivo* phagocytic activity of AM during a T cell-mediated immune response by quantifying the percentage of AMs that contain internalized Pc. Understanding the mechanisms controlling Pc phagocytosis is an area of great interest, and many investigators have utilized various techniques to perform *in vitro* assessments of Pc phagocytosis [63]–[70]. However, demonstrating an *in vivo* role for AM phagocytosis in the clearance of Pc has been more difficult. AM with associated Pc have been observed in the BAL fluid of patients and animals [71]–[74]. However, this was in the setting of active PcP, the level of phagocytosis appeared low, and the significance to organism clearance was not determined. Others have performed *in vivo* assessments of phagocytosis immediately (within 24 hours) following a bolus inoculation of labeled Pc [75], [76]. In addition, short-term depletion of AMs in rats reduced the clearance of Pc over the initial 24 hours post-inoculation [77]. While these studies were able to demonstrate a role for AM *in vivo*, the timing indicates that the investigators were evaluating the innate immune response to a bolus inoculation of Pc, rather than the CD4^+^ T cell-mediated response which is required for natural clearance of Pc from the lung. Using this new technology we were able to develop an assay to show that AMs are effector cells for the clearance of Pc during a natural CD4^+^ T cell-mediated immune response *in vivo*. The advantages of these ImageStream-based data are that: 1) internalized Pc was distinguished from attached Pc; 2) a large number of AM from each animal was rapidly assessed to provide quantification of the phagocytic response; 3) the dependence of phagocytic activity on the presence of CD4^+^ T cells was demonstrated; and 4) the CD4^+^ T cell-dependent increase in phagocytic activity correlated with the clearance kinetics of Pc. Importantly, the ImageStream data was validated using confocal microscopy to co-localize intracellular Pc with the lysosome protein LAMP-1. These data indicate that Pc is located within the phagolysosome of AM, consistent with phagocytosis of the pathogen. The multispectral imaging flow cytometry technology should provide a valuable tool for further study of Pc phagocytosis *in vivo*.

In summary, the results of this study indicate that the immune response to Pc can be modulated in a manner that reduces inflammatory consequences of PcP while enhancing the pathogen clearance through increased AM phagocytic capacity. We also developed a method for *in vivo* quantification of AM phagocytosis of Pc, and provide evidence that the macrophage is the ultimate effector for the CD4^+^ T cell-mediated clearance of Pc from the lungs. Immune modulation of T cell and AM functions should be considered potential therapeutic targets for the treatment of immune complications of PcP. Macrophages are equipped to recognize and eliminate pathogens as well as promote and/or resolve inflammation. Our results indicate that the phagocytic function of macrophages can be enhanced with a concomitant reduction in their pro-inflammatory properties. Enhancement of AM-mediated clearance of Pc may prove less inflammatory and generally superior to antibiotic therapy alone.

## Methods

### Pc source animals

Severe combined immunodeficient (SCID) mice on a C.B-17 background (C. B-Igh-1^b^/Icr Tac-Prkdc^scid^) were purchased from Taconic (Hudson, NY), or obtained from a breeding colony at the University of Rochester. The mice were housed using micro-isolator technology and fed sterilized food and water. To induce infection SCID mice were co-housed with Pc-infected SCID mice. Pc organisms were isolated as previously described [38]. Pc cysts were enumerated by standard Gomori's methenamine silver stain.

### Sulfasalazine and trimethoprim-sulfamethoxazole administration

Sulfasalazine (SSZ) (Sigma, St. Louis, MO) was administered once daily by intra-peritoneal (i.p.) injection at a dose of 200 mg per kg of body weight. Trimethoprim Sulfamethoxazole (TMP-SMX) (SICOR Pharmaceuticals, Inc. Irvine, CA) was administered once daily i.p. at a dose calculated to give 16 mg per kg of body weight of the Trimethoprim component of the drug combination. This dose was based on the therapeutic dose given to humans for the treatment of PcP.

### Mouse model of PcP-related IRIS

To induce infection SCID mice were intra-nasally inoculated with 1×10^5^ purified Pc based on cyst count. Three weeks later the mice were immunologically reconstituted with 5×10^7^ congenic spleen cells from normal C.B-17 mice.

### *In vivo* CD4^+^ T cell depletion

CD4^+^ T cells were depleted by i.p. injection of monoclonal antibody specific for mouse CD4 (clone GK1.5, ATCC TIB207). Antibody injections (250 µg per mouse) were given one day prior to and one day after immune reconstitution. Thereafter, antibody was administrated every four days for the duration of the experiment.

### Physiologic assessment of pulmonary function in live, ventilated mice

Lung compliance and resistance were measured in live ventilated mice using a whole body plethysmograph (BUXCO Electronics Inc., Wilmington, NC) connected to a Harvard rodent ventilator (Harvard Apparatus, Southnatic, MA) as previously described [78]. Dynamic lung compliance was normalized to the peak body weight of the animal. Respiratory rates were measured using whole body unrestrained chambers (BUXCO Electronics Inc). Data was collected and analyzed using the Biosystems XA software package (BUCXO Electronics Inc.).

### Bronchoalveolar lavage (BAL) and lung tissue preparation

Right lung lobes were lavaged with four, one-ml aliquots of 1X Hank's balanced salt solution. Cell-free lavage fluid (approximately 3.5 ml per mouse) was frozen at −80°C. BAL cells were enumerated, and then differentials and multi-parameter flow cytometric analyses were performed. Anti-CD4-Fluorescein (clone RM4-4) and anti-CD8a-Peridinin Chlorophyll-α Protein (clone 53-6.7), were purchased from BD Biosciences (San Diego, CA). The anti-CD4 clone RM4-4 was used to confirm CD4^+^ cell depletion *in vivo* because it is not blocked by the CD4-depleting antibody (clone GK1.5). Cells were analyzed on a FACSCalibur (BD Biosciences, San Jose, CA), with at least 10,000 events routinely analyzed for each Pc-infected mouse. At least 5,000 events were analyzed from uninfected control mice.

For fixation the lungs were inflated with 15 cm gravity flow-pressure of 10% formalin (Sigma, St. Louis, MO). The lungs were fixed in situ for 10 minutes under gravity flow pressure, and then removed from the animal and placed in fixative for 16 h at 4°C. Lung tissue was embedded in paraffin and 4 µM sections were cut. Hematoxylin and eosin was used to visualize tissue.

### Cytokine ELISAs of BAL fluid

Total protein concentration was determined in cell-free lavage by the colorimetric assay of Lowry. Albumin concentration was determined using the Mouse Albumin ELISA Quantitation kit from Bethyl Laboratories (Montgomery, TX). TNF-α, IFN-γ, MCP-1, and RANTES ELISA kits were used according to the manufacturer's instructions (R&D, Minneapolis, MN).

### Real-time PCR assessment of lung Pc burden

Since Pc cannot be cultured, a real-time PCR method was used to quantify lung burden. For quantification of Pc burden in right lung lobes, quantitative PCR using TaqMan primer/fluorogenic probe chemistry and an Applied Biosystems Prism 7000 Sequence Detection System (Applied Biosystems, Foster City, CA) was performed with a primer/probe set specific for the mouse Pc kexin gene as previously described [78].

### *In vivo* assessment of macrophage phagocytosis of Pc using ImageStream

For quantitation of Pc phagocytosis, an ImageStream multispectral imaging flow cytometer (Amnis Corporation, Seattle, WA) was used [79], [80]. With this technology the number of BAL AM with internalized Pc was directly quantified. CD11c was used as a surface marker to identify AM, while anti-Pc antibodies were used to stain internalized Pc. Whole lungs were lavaged and BAL cells were washed with ice cold PBS with 1%BSA (PBA), and incubated with mouse Fc Block (BD Biosciences, San Diego, CA) for 5 min on ice. Cells were then surface stained with anti-CD11c-phycoerythrin (clone HL3, BD Biosciences) for 30 minutes on ice and washed with PBA. The cells were then permeabilized with BD Cytofix/Cytoperm Fixation and Permeabilization Solution (BD Biosciences), and incubated with a pool of five different anti-*Pneumocystis* monoclonal antibodies for 30 minutes on ice. These antibodies were generated in our laboratory and were chosen because they recognize five different epitopes on the surface of Pc as determined by western blot and IFA (4F11, 2B5, 3D6, 1F1, 1F5). Characterization of antibody 4F11 has been published [81], but the remaining antibodies have not been further characterized. Following a wash step, the cells were incubated with Alexa Fluor 647 goat anti-mouse IgG (H+L) (Invitrogen Molecular Probes, OR) for 30 minutes on ice. Stained cells were washed, pelleted, and resuspended in 50 µl of ice cold 1% paraformaldehyde in PBS (Electron Microscopy Sciences, PA). Samples were stored at 4°C in the dark until analyzed. Twenty thousand to forty thousand event image files were collected for each sample on an ImageStream100 using 200 mW of 488 nm and 90 mW of 658 nm laser power.

The data obtained were analyzed using the ImageStream Data Exploration and Analysis Software (IDEAS, Amnis), which quantifies morphometric and photometric parameters on a per-cell basis for large populations of collected events [79], [82]. Single AM cells were gated as those events with normal brightfield area, high brightfield aspect ratio and CD11c positive staining. Each analyzed file contained at least 5000 AM events, enabling routine statistical analysis. AM that had phagocytosed Pc were identified by gating on CD11c+ events with high Pc-AF647 Max Pixel values (discriminates punctuate Pc from diffuse background staining and autofluorescence) and high Pc Internalization values. The Internalization score is a ratio of the intensity of bright red staining inside the cell (defined by eroding the CD11c mask 6 pixels) to the bright red staining in the membrane (defined by subtracting the latter mask from the CD11c mask dilated 3 pixels), and the higher the score the greater the concentration of Pc inside the cell. In order to condition the measurement to Pc particles in sharp focus (and thus in the central focal plane of the cell), only the mean of the upper quartile pixel intensities, weighted by the max pixel intensity, is used to compute the ratio [83]. The percentage of AM with internalized Pc was derived directly from the ImageStream data. The absolute number of AM with internalized Pc that was recovered from each animal was calculated by multiplying this percentage by the total number of AM recovered.

### Confocal fluorescence microscopy

Cells were stained with the identical antibodies under the identical conditions described for ImageStream analyses with the following exceptions. Anti-mouse CD11c-AlexaFluor 488 (Invitrogen Molecular Probes, OR) was used instead of CD11c-PE. Also, in some experiments, biotinylated anti-mouse CD107a (LAMP-1) (Biolegend, CA) was used to co-localize intracellular Pc with lysosomes. Cells were first stained with anti-CD11c and anti-Pc antibodies as described above. After a wash and second permeabilization step, cells were stained with biotinylated anti-CD107a antibody for 30 minutes on ice followed by streptavidin conjugated with PE-Texas Red (BD Bioscience, CA). After fixation, cells were centrifuged onto glass slides, mounted with anti-fade Vectashield (Vector Laboratories, CA), and coverslipped for optimal imaging. Cells were imaged using an FV1000 Olympus Laser Scanning Confocal Microscope using an I×81 inverted microscope and a 60× objective with zoom of 4. Lasers used were 405, 488, 559, and 635 optimized to reduce photo-bleaching and used sequentially. Differential Interference Contrast (DIC) was performed using the 559 laser. Pixel dwell times were 8 us/pixel and 1024×1024 pixel format for high resolution imaging. Parameters were maintained consistent throughout imaging. All the images presented are the originals without post-processing.

### Analyses of alveolar macrophage phenotype

BAL cells were collected and centrifuged onto glass slides. Cells were fixed with 3% paraformaldehyde and initially stained with hamster anti-mouse CD11c (Abcam, MA) followed by either goat anti-hamster AF594 or goat anti-hamster AF488 (Invitrogen Molecular Probe, Oregon) secondary antibody. After permeabilization with 0.2% Triton ×−100 in phosphate buffered saline, cells were stained with rabbit anti-mouse iNOS (Abcam, MA) with goat anti-rabbit AF488 (Invitrogen Molecular Probe, Oregon) secondary antibody, or goat anti-mouse Arginase (Santa Cruz Biotechnology, CA) with donkey anti-goat AF546 (Invitrogen Molecular Probe, Oregon) secondary antibody. Slides were mounted with anti-fade Vectashield (Vector Laboratories, CA) and coverslipped for optimal imaging. A Nikon Eclipse E400 fluorescence microscope was used for photomicroscopy. All photographs for a given protein were taken with identical exposure settings. The ImageJ software (National Institutes of Health) was used to quantify the mean fluorescent intensity of iNOS and Arginase staining in CD11c positive alveolar macrophages.

### Statistical analyses

One-way analysis of variance was performed with the SigmaStat 2.0 software (Jandel, San Rafael, Calif.). The Student-Newman-Keuls method was used for all pair-wise multiple comparisons.

### Ethics statement

All animal protocols were pre-approved by University Committee on Animal Resources (UCAR) at the University of Rochester Medical Center according to the guidelines of the Association for Assessment and Accreditation of Laboratory Animal Care International.
